# A Second Non-prosthetic Peri-Implant Femoral Fracture (PIFF) Following Plating for a Primary PIFF After a Trochanteric Femoral Fracture

**DOI:** 10.7759/cureus.95485

**Published:** 2025-10-27

**Authors:** Hiroki Yamada, Tomohiro Yoshizawa, Akihito Suto, Takeshi Makihara, Katsuya Aoto

**Affiliations:** 1 Orthopedic Surgery, Kasumigaura Medical Center, Tsuchiura, JPN; 2 Orthopedic Surgery, Institute of Medicine, University of Tsukuba, Tsukuba, JPN

**Keywords:** hip fracture, locking attachment plate, osteoporosis, peri-implant femoral fracture, trochanteric fracture

## Abstract

Due to the increasing number of patients with osteoporosis, proximal femoral fractures are on the rise in Japan's super-aging society. With the widespread adoption of surgical treatment and improved prognosis, the number of patients surviving for a long time after surgery has increased, and the occurrence of non-prosthetic peri-implant femoral fractures (PIFFs) is also trending upward. To our knowledge, there have been no previous case reports of a second PIFF after an operation for a PIFF. We report a case of an 83-year-old woman who sustained a second PIFF after surgery for a trochanteric femoral fracture. The initial fracture was treated with a short cephalomedullary nail. Although a bone union from the trochanteric fracture was obtained, a subsequent PIFF distal to the nail occurred and was fixed with a lateral femoral plate and cables. However, a second PIFF occurred again distal to the plate. In the surgery for the second PIFF, we used a long distal femoral plate that covered the entire femur along with a locking attachment plate (LAP). This achieved a strong fixation, and the patient had a good postoperative course, with bone union confirmed at four months, and she became able to walk with a cane. This case suggests that the choice of treatment for the first PIFF influences the risk of subsequent refracture. The LAP used in this case is considered a useful treatment option for preventing a second PIFF, as it allows for strong fixation while preserving the existing implant. In a super-aging society where the possibility of multiple PIFF is increasing, it is important to carefully select the initial treatment.

## Introduction

In Japan's super-aging society, the number of patients with osteoporosis is increasing, and the number of patients with proximal femoral fractures continues to rise. Proximal femoral fractures are considered a serious social problem as they significantly reduce quality of life (QOL) and activities of daily living (ADL) [1] and increase mortality [2]. Surgical treatment is common for proximal femoral fractures. While intramedullary nailing has become the standard treatment in recent years, plating is also used. Due to increased life expectancy and improved outcomes of proximal femoral fracture treatment, a growing number of patients maintain their ADL and survive long-term after surgery. It has been suggested that non-prosthetic peri-implant femoral fractures (PIFFs) are on the rise as a complication [3-7]. PIFFs are defined as a femoral fracture in the presence of a pre-existing non-prosthetic implant. PIFFs represent a distinct pathology from periprosthetic fractures. The incidence of PIFFs after cephalomedullary nail fixation has been reported to be 0.45-2.1% [3-7]. Despite the increasing number of patients with PIFFs, not much literature has been published to date. We report on a case of a supracondylar femoral PIFF that occurred after plate fixation for a PIFF following proximal femoral fracture surgery.

## Case presentation

An 83-year-old woman whose ADL is at the level of walking with a cane presented to the emergency department with pain and deformity of the left distal thigh following a traffic accident. Her medical history was notable for hypertension, hyperlipidemia, and osteoporosis.

**Figure 1:**
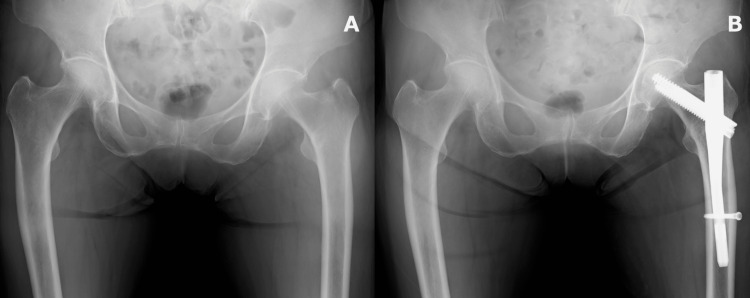
Radiograph findings A: preoperative anteroposterior radiograph; B: postoperative anteroposterior radiograph

At the age of 76, she sustained a trochanteric femoral fracture from a ground-level fall at home, which was treated with closed reduction and internal fixation (CRIF) using a short cephalomedullary nail (TRIGEN INTERTAN; Smith & Nephew, Memphis, Tennessee) (Figure 1).

**Figure 2:**
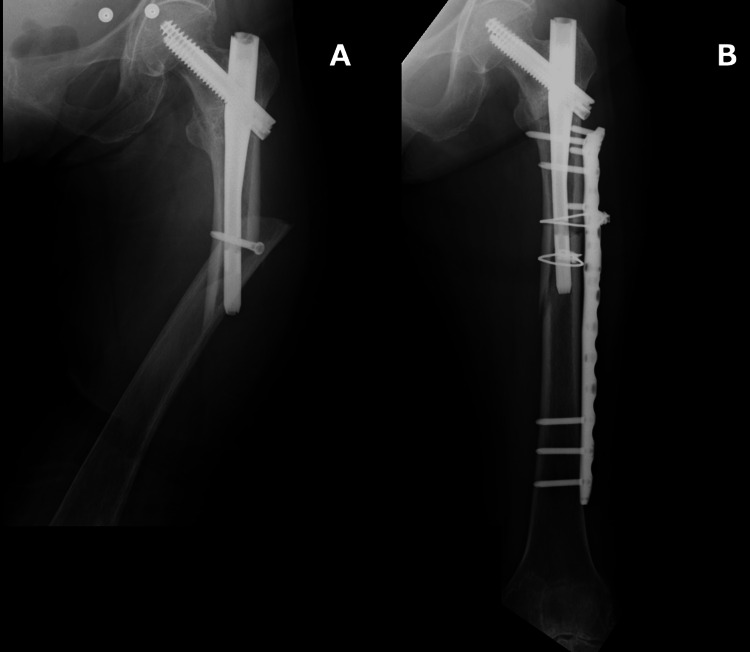
Radiograph findings A: preoperative anteroposterior radiograph; B: postoperative anteroposterior radiograph

At the age of 77, she sustained another ground-level fall at home, which resulted in a PIFF. At this time, the bony union of the initial femoral trochanteric fracture was confirmed postoperatively. This fracture was treated with open reduction and internal fixation (ORIF) using the NCB Periprosthetic Proximal Femur Plate (Zimmer Biomet, Winterthur, Switzerland) (Figure 2). She recovered well from her first PIFF and, by the second postoperative month, regained ambulation with a cane; however, range of motion (ROM) was limited (90° flexion, -10° extension) due to underlying knee osteoarthritis.

Regarding osteoporosis, the young adult mean (YAM) values at the time of the first trochanteric femoral fracture were 79% for the lumbar spine and 62% for the femur. At that point, treatment with active vitamin D and a monthly bisphosphonate (BP) was initiated. However, following the first PIFF, the regimen was changed to a parathyroid hormone preparation, and the management of osteoporosis was transferred to the patient's primary care physician. The final YAM values recorded at the age of 79 were 86% for the lumbar spine and 66% for the femur. Subsequently, the treatment was modified to an oral monthly BP formulation, and this therapy has been continued to the present. 

**Figure 3:**
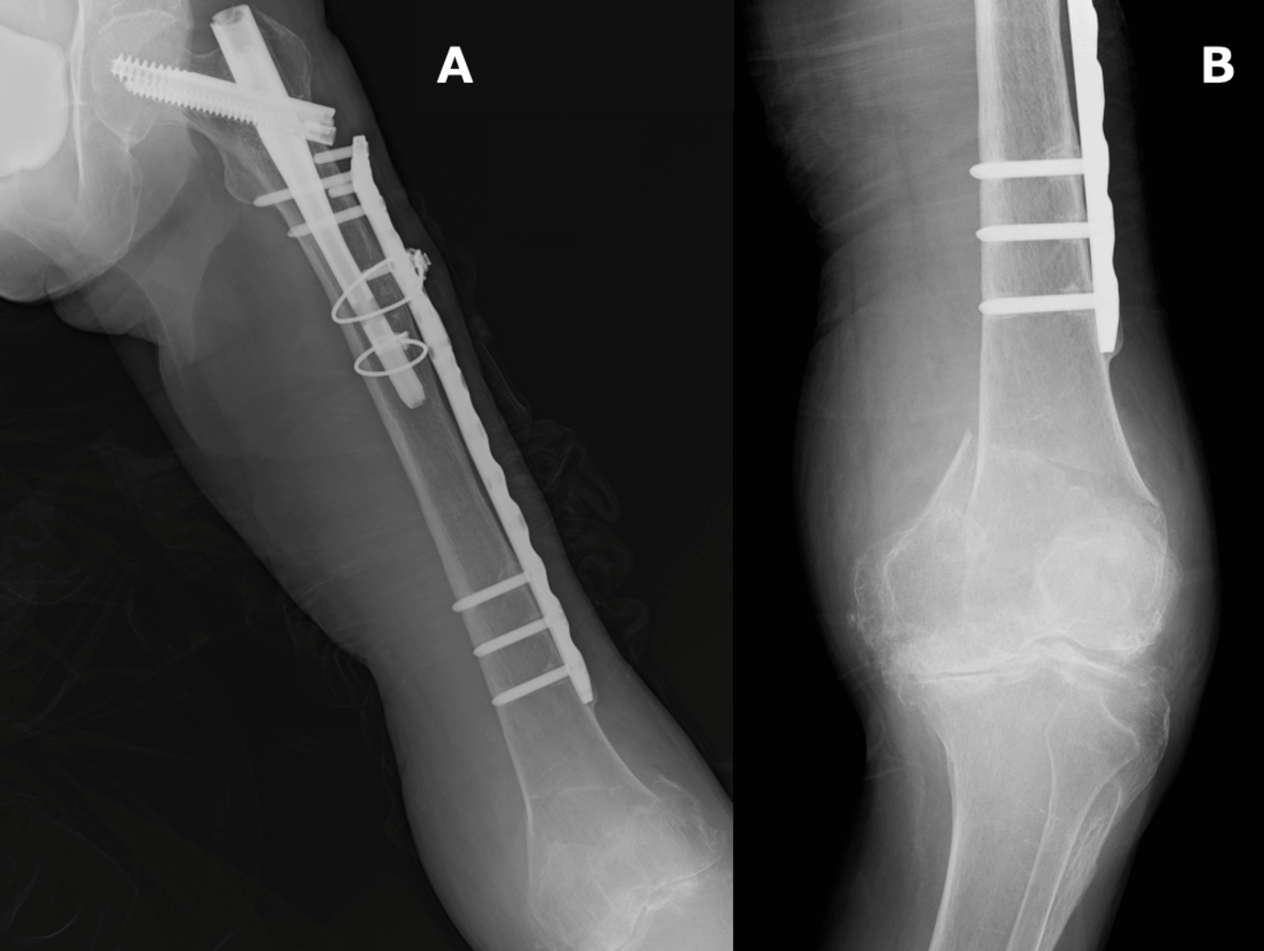
Radiographic examinations A: preoperative full-length anteroposterior radiograph of the femur; B: preoperative anteroposterior radiograph of the knee

At the age of 83, she sustained an injury to her left thigh in a traffic accident while in the passenger seat. Radiographic examinations revealed a supracondylar PIFF distal to the pre-existing femoral lateral plate (Figure 3).

**Figure 4:**
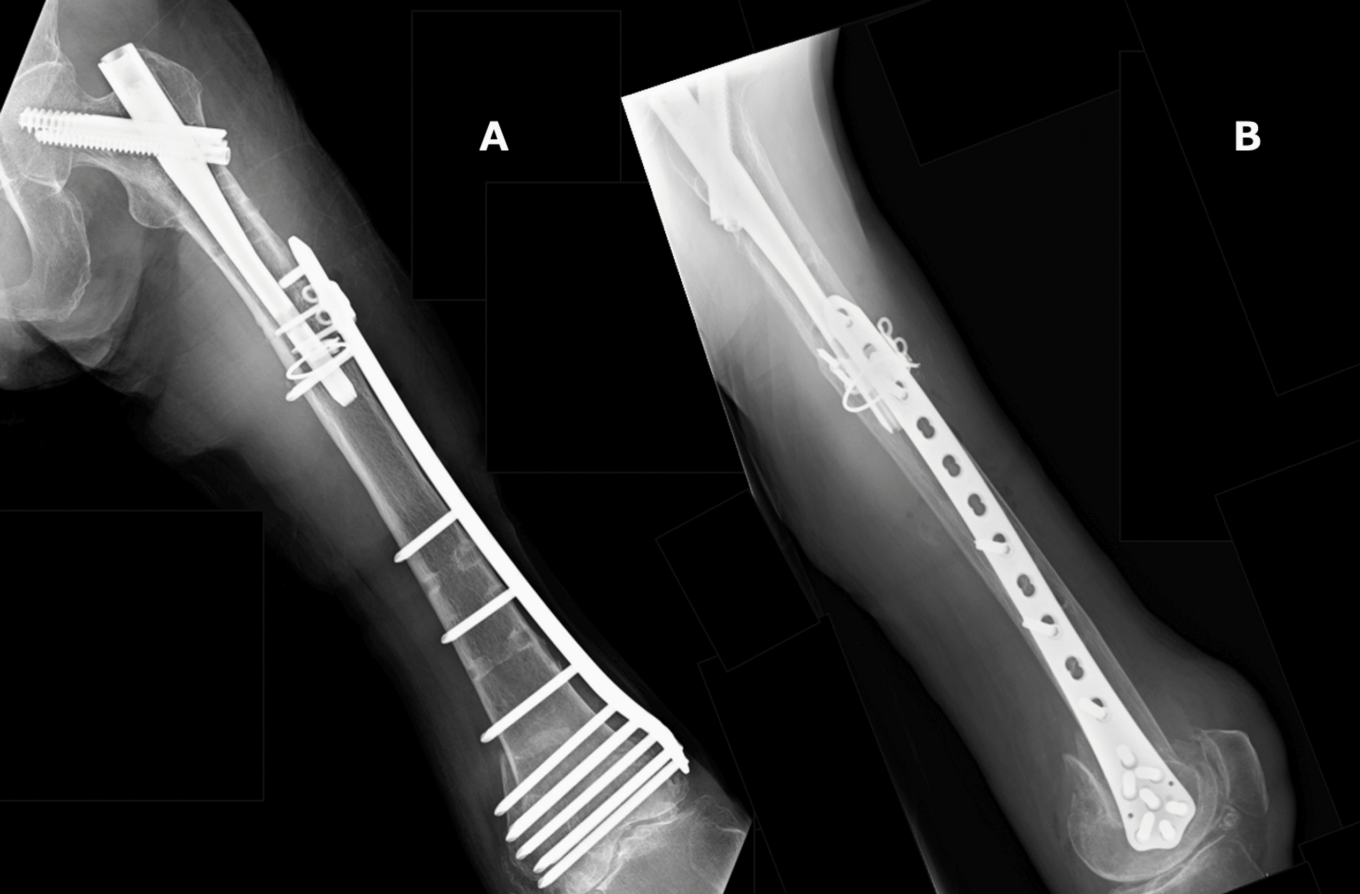
Radiograph findings A: postoperative anteroposterior radiograph; B: postoperative lateral radiograph

**Figure 5:**
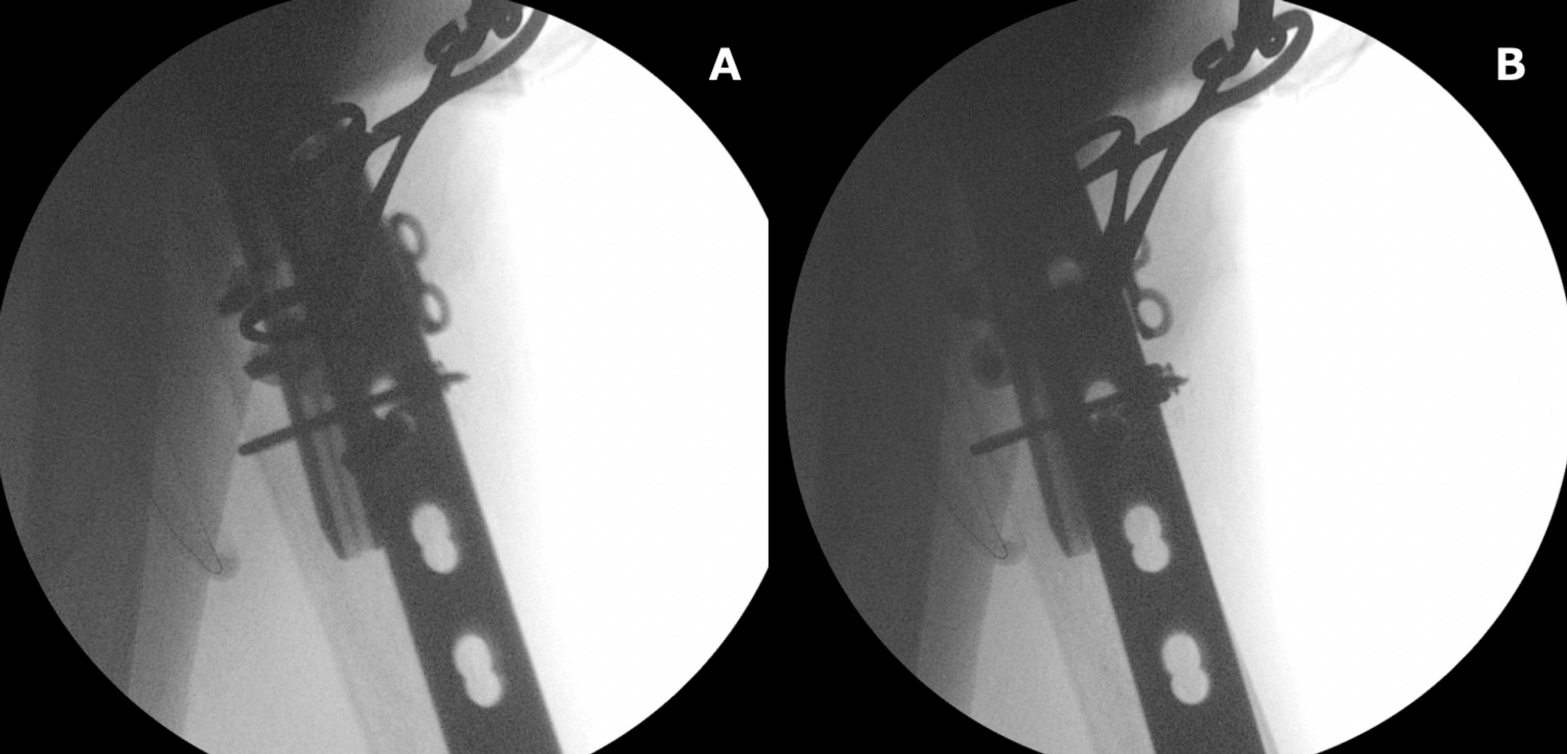
Intraoperative radiographs depicting the screw has been inserted into the bicortex A: lateral view; B: lateral view with a slightly different angle

The pre-existing NCB Periprosthetic Proximal Femur Plate was explanted, and a locking compression plate-distal femur (LCP-DF; DePuy Synthes, Solothurn, Switzerland) was used for fixation, ensuring an overlap of greater than 6 cm with the short cephalomedullary nail [8]. The locking attachment plate (LAP, Depuy Synthes, Solothurn, Switzerland) was attached to the second most proximal screw hole. Two bicortical screws were then inserted through the posterior tab, carefully avoiding the existing implant. A monocortical periprosthetic screw was inserted into the most proximal hole, and a bicortical locking screw was inserted into the third most proximal screw hole (Figure 4). Intraoperative fluoroscopy confirmed bicortical screw insertion from the LAP, thus achieving strong fixation from the distal femur to the implant overlap zone (Figure 5).

**Figure 6:**
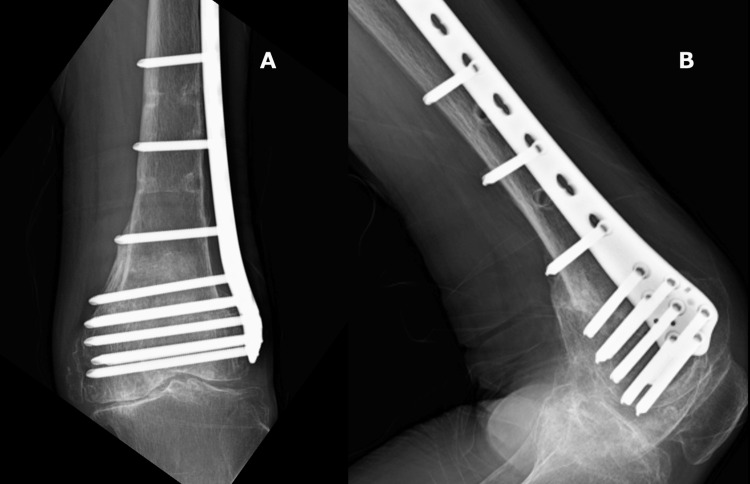
Postoperative radiographs at four-month follow-up A: anteroposterior view; B: lateral view

Following a six-week period of non-weight-bearing, the patient began weight-bearing as tolerated. She was able to ambulate with a cane at two months postoperatively and was subsequently discharged. Radiographs showed that bony union was achieved at four months postoperatively (Figure 6). Although her knee ROM remained limited (90° flexion, −10° extension), this finding was consistent with her preoperative status. At the four-month follow-up, she was ambulating with a cane without any signs of instability.

## Discussion

A PIFF is defined as a femoral fracture in the presence of a pre-existing non-prosthetic implant. PIFFs represent a distinct pathology from periprosthetic fractures. Although the incidence of PIFFs tends to increase, the number of reported cases appears to be relatively low.

Chan et al. [9] proposed a classification system for PIFFs that considers three key factors: the type of implant originally used (plate or nail), the status of the initial fracture at the time of the PIFF (union, non-union, or failure (subtypes A, B, or C)), and the location of the new fracture relative to the implant's position (Type 1 for fractures at the distal implant tip or Type 2 for more distal locations).

Building on Chan's classification, Bidolegui et al. [10] examined a detailed treatment algorithm for PIFFs where the initial fracture has healed (Chan's classification subtype A). For fractures at the distal tip of a short cephalomedullary nail, as in this case (N1A*s), they recommend converting to a long cephalomedullary nail. In contrast, for fractures located more distally than a short cephalomedullary nail (N2A*s) or at the distal end of a long cephalomedullary nail (N1A*l), they recommend using a distal femoral plate with an overlap of at least twice the medullary canal's diameter. This approach creates a biomechanically stable construct, which promotes bony union and reduces the risk of a subsequent fracture occurring in a stress riser [11].

Based on the application of the above Chan’s classification and Bidolegui's treatment strategy to this case, the classification was N1A*s, and the recommended initial treatment was conversion to a long nail. Conversion to a long nail allows for coverage of the entire femur, thereby preventing secondary fractures resulting from stress concentration and ensuring stable fixation. Although utilizing a long nail would have been a more desirable treatment option for the initial PIFF, this approach is not without limitations. Moreover, various risks are associated with nail removal, including subsequent fractures, bleeding, and difficulty in removing the implant itself. Goodnough et al. [12] specifically reported that converting to a long cephalomedullary nail for fractures at the tip of a short nail (corresponding to Chan’s classification N1A*s) carries the risk of increased intraoperative and postoperative complications.

However, ORIF with plate fixation was the initial method selected in the current case. While ORIF offers the advantage of not requiring implant removal, it necessitates both implant overlapping and full coverage of the entire femur. Furthermore, ensuring stability at the implant's overlapping zone typically requires the use of monocortical screws in that region. To further enhance construct stability and prevent screw backout, wiring is often employed in conjunction with the plate.

It is inferred that the surgeon chose the NCB Periprosthetic Proximal Femur Plate to maximize fixation around the existing nail by utilizing its multiple screw holes. However, a plate of sufficient length to extend to the femoral condyle and cover the entire femur was not utilized, a decision likely related to the distance from the nail tip. The primary shortfall was that the basic principle of PIFF, achieving a biomechanically stable construct, was compromised by the failure to achieve full femoral coverage.

Therefore, although the initial plate fixation may have been sufficient for bony union, full coverage of the femur would have been a more ideal approach. Crucially, either of these alternative strategies, conversion to a long nail or the use of a full-length plate, would likely have prevented the occurrence of the second PIFF.

The second PIFF was treated with the LCP-DF and the LAP. It has been reported that bicortical screw fixation provides greater mechanical stability than a combination of a plate and wiring when treating a fracture with an overlapping implant [13]. By attaching the LAP to an LCP, bicortical screws can be inserted lateral to the LCP screw holes, effectively bypassing the intramedullary implant. Global adoption of the LAP began in 2009, with clinical results appearing in the literature since 2012 [14], while its use commenced in Japan in 2021.

The advantage of the LAP is that it provides greater mechanical stability than a combination of a plate and wiring [13]. A potential disadvantage, however, is that the number of bicortical screws that can be inserted may be constrained by the diameter of the existing implant, potentially compromising fixation strength. As treatment for the second PIFF, we chose the LCP-DF and the LAP. ORIF was performed, covering the femoral condyle and ensuring an overlap of at least twice the medullary canal's diameter in the implant area.

The non-weight-bearing period was prolonged compared to typical intramedullary nail treatment. The patient's ADLs and walking ability were maintained with appropriate rehabilitation, and she regained the ability to walk independently. Bony union was achieved at four months postoperatively, and the patient had a favorable outcome.

Despite the recommendation by Bidolegui et al. [10] to convert to a long cephalomedullary nail for the initial PIFF, the recent introduction of the LAP now offers a novel and effective alternative: ORIF with an LCP that spans the entire femur, augmented by the LAP. Although not much literature on second PIFFs has been published to date, in the current case, we explanted the NCB Periprosthetic Proximal Femur Plate and chose the LCP-DF, which covered the entire femur. Sufficient fixation was achieved by utilizing the LAP in the implant's overlap region, which led to bony union and a favorable outcome.

## Conclusions

In super-aged societies such as Japan, the risk of multiple PIFFs is increasing. Accordingly, to prevent a second or subsequent PIFF, the treatment strategy for the initial PIFF should be selected carefully. The LAP is useful in enhancing fixation strength in areas overlapping with pre-existing implants, thereby broadening the range of available treatment options for this challenging condition.
